# A comparative review of deep and spiking neural networks for edge AI neuromorphic circuits

**DOI:** 10.3389/fnins.2025.1676570

**Published:** 2025-10-02

**Authors:** Pietro M. Ferreira, Siqi Wang, Yueyuan Gao, Aziz Benlarbi-Delai

**Affiliations:** ^1^University Savoie Mont Blanc, University Grenoble Alpes, Grenoble INP, CNRS, CROMA, Chambéry, France; ^2^Sorbonne Université, CNRS, Laboratory de Génie Électrique et Électronique de Paris, Paris, France; ^3^Université Paris-Saclay, CentraleSupélec, CNRS, Laboratory de Génie Électrique et Électronique de Paris, Gif-sur-Yvette, France; ^4^Faculty of Materials for Energy, Shimane University, Matsue, Japan

**Keywords:** energy efficiency, neuromorphic circuits, edge AI, spiking neural network (SNN), deep neural networks (DNN)

## Abstract

Edge AI implements neural networks directly in electronic circuits, using either deep neural networks (DNNs) or neuromorphic spiking neural networks (SNNs). DNNs offer high accuracy and easy-to-use tools but are computationally intensive and consume significant power. SNNs utilize bio-inspired, event-driven architectures that can be significantly more energy-efficient, but they rely on less mature training tools. This review surveys digital and analog edge-AI implementations, outlining device architectures, neuron models, and trade-offs in energy (J/OP), area (μm^2^/OP), and integration technology.

## 1 Introduction

Neuromorphic computing emerged in the 1990s as a complement to von Neumann architectures, exploring bio-inspired neural systems. With the rise of Internet of Things (IoT) applications, neural networks (NNs) are increasingly implemented directly in hardware, known as edge AI. Choosing the right NN architecture is nontrivial: while hardware design is driven by power, area, and speed, NN performance depends on training methods, architecture, and hyperparameters.

Deep neural networks (DNNs) have achieved remarkable success (Li et al., 2022), leveraging backpropagation with optimizers such as SGD and Adam. Supported by mainstream libraries like TensorFlow (Hope et al., 2017), they are accessible but computationally intensive and not suitable for edge AI. Such a solution presents an energy efficiency consideration in terms of the number of floating operations per second (i.e., *E*_*eff*_ in W/FLOPS), and it is often implemented in cloud computing. More efficient edge implementations exist in microcontrollers, such as TinyOL (Ren et al., 2021), TinyTL (Cai et al., 2020), MCUNet (Lin et al., 2020), STM32N6 (El-Ouazzane, 2024), if model compression and limited accuracy are considered. Such solutions enable frugal AI with milliwatt-level power.

Spiking neural networks (SNNs) bridge artificial and biological intelligence on low-power devices (Shrestha et al., 2022). State-of-the-art digital implementations include SpiNNaker (Furber et al., 2014), TrueNorth (Debole et al., 2019), and Loihi 2 (Orchard et al., 2021). Learning rules such as spike-time-dependent plasticity (STDP) in Gautam and Kohno (2021) support bio-inspired applications, but neuromorphic chips remain niche due to cost and availability. Analog SNNs mimic biological neurons with excellent energy efficiency, down to fJ/SOP in Danneville et al. (2019), but face challenges in depth, silicon integration, reliability, and training tools compared to digital solutions.

The widespread topic discussion and the variety of experimental conditions are a challenge for a systematic literature review. This review compares digital and analog edge-AI approaches, focusing on neuron models, device architectures, and trade-offs in energy (J/OP), area (μm^2^/neuron), and integration technology. As benchmarks remain fragmented, we highlight challenges and opportunities across both domains. To the best of the author's knowledge, this review is the first work comparing NN solutions of neuromorphic circuits for edge AI, converging both points of view.

## 2 Deep neural network

Conventional neural network (NN) architectures consist of recurrent, convolutional, pooling, and fully connected layers selected to solve classification, regression, or generative problems. They are highly effective for regression, classification, clustering, modeling, segmentation, control or decision-making, generative, and ranking or recommendation problems. Depending on the architecture and the problem, many names have been used to describe the NN. Indeed, an NN is made up of neurons, and the most common mathematical model of neurons is the McCulloch and Pitts (Li et al., 2022). Perceptron models were developed, including learning capabilities for such neurons. Feedforward and backpropagation algorithms became popular in NN training while using SGD and tailored loss functions. According to Li et al. (2022), challenges such as local optima, overfitting, gradient vanishing, and gradient exploding were responsible for the paradigm shift to DNNs. DNNs are characterized by (i) multiple hidden layers and (ii) layer-wise pre-training. Thus, the name DNN has become the most widely used term in the literature to describe a layered computational model composed of multiple interconnected layers of neurons capable of extracting and representing complex patterns from input data.

DNNs leverage different activation functions [*f*(·)] to express the complex non-linear capabilities of a neuron. Common *f*(·) functions are sigmoid, hyperbolic tangent, Swish, Mish, and rectified linear unit functions, along with their variations. The data input (*x*_*i*_) is multiplied by synaptic weights (ω_*i, j*_), which are trainable variables in an NN, and then a bias (*b*_*j*_) is added to represent the internal state of neuron (*j*). The data output (*y*_*j*_) of layer *i* is mathematically described as *y*_*i*_ = *f*(∑*x*_*i*_ · ω_*i, j*_ + *b*_*j*_). To capture the discrepancy between a mathematical model's predictions and the observed data, a figure of merit reflecting the model error is often referred to as an objective or a loss function. The most commonly used loss functions are the mean squared error, the mean absolute error, and the cross-entropy loss, applied to binary, categorical, or logit-based probabilities. The use of an optimization algorithm is the best solution to iteratively update the set of ω_*i, j*_ in a way that decreases the loss over time (or epoch).

Lemma 1. If *ŵ*_1_ = *ŵ*_0_ − γ∇*F*(*ŵ*_0_), where ∇*F*(*ŵ*_0_) is the gradient of *F* evaluated at *ŵ*_0_, then for a small enough γ,


$$\begin{array}{l} F\left({\hat{w}}_{0}\right)\geq F\left({\hat{w}}_{1}\right). \end{array}$$

While convergence to the global minimum is guaranteed for convex functions, optimization in non-convex settings (e.g., typical in DNNs) may lead to convergence on local minima. Nevertheless, in practice, such solutions are often sufficient, as demonstrated by the remarkable empirical success of DNNs.

Unlike full-batch gradient descent, which computes gradients using the entire dataset, SGD estimates gradients using mini-batches, typically comprising 50 to 500 samples. While smaller mini-batches reduce computation time per update, they introduce higher variance in gradient estimates, leading to fluctuations in the objective function. These fluctuations, though potentially destabilizing, can aid convergence in non-convex landscapes by helping the optimizer escape shallow local minima. As a result, mini-batch SGD has become standard in DNN training. A critical hyperparameter is the learning rate, which governs the step size along the negative gradient. If set too high, the optimizer may diverge; if too low, convergence may be excessively slow.

The Adam algorithm is a gradient-based optimization algorithm that improves upon standard SGD by incorporating momentum and adaptive learning rates. Unlike SGD, which applies a single global learning rate, Adam maintains per-parameter learning rates that adapt during training based on estimates of the first and second moments (i.e., the mean and uncentered variance) of the gradients. This allows Adam to efficiently handle sparse gradients and noisy data. Moreover, it is more stable and converges faster, especially in high-dimensional, non-convex optimization. As a result, Adam often requires less hyperparameter tuning and performs well out of the box across a wide range of deep learning tasks.

Cloud computing implementation of DNNs is outside the scope of this review, and learning TensorFlow is a great reference for delving into this subject, as in Hope et al. (2017). Considering edge computing implementation, DNNs can be implemented in graphics processing units (GPUs), microcontrollers, and field-programmable gate arrays (FPGAs). GPUs are commonly used to accelerate deep learning processes, where NNs are online trained through hardware accelerators (Rothmann and Porrmann, 2022). They are the main solution, as area costs are low and deployment solutions are fully compatible with both cloud computing environments. GPU-accelerated architectures ensure NN scalability, high-performance processing, and efficient resource utilization. The main drawback is power consumption, which ranges from hundreds of watts to thousands of watts. Tiny machine learning and frugal AI architectures are a fast-growing research area committed to democratizing deep learning for all-pervasive microcontrollers (Ren et al., 2021). The challenges are the power, memory, and computation limitations of microcontrollers. However, such solutions are based on batch/offline settings, and they support only the NN's inference on microcontrollers. FPGAs provide flexible, distributed on-chip memory resources, such as LUT-based distributed RAM and dedicated memory. These resources enable the design of domain-specific architectures, resulting in high computational speed, less data movement, and improved energy efficiency compared to microcontrollers (Rothmann and Porrmann, 2022). The literature shows that FPGA-based implementations can achieve performance gains comparable to those of GPU-based implementations for the specific workloads tested. Nevertheless, microcontrollers remain a popular low-cost, low-power solution for hardware-friendly NNs.

Cai et al. (2020) have researched memory-efficient on-device learning solutions for microcontroller implementation. The work of Cai et al. (2020) proposes to freeze the weights while only learning the bias modules, which reduces the storage required for the intermediate activations. Lin et al. (2020) has proposed a system-model co-design framework that enables deep learning on off-the-shelf microcontrollers. The proposal is a two-stage neural architecture search capable of handling the tiny and diverse memory constraints. Ren et al. (2021) have proposed a novel system called TinyOL, including incremental online on-device learning capabilities. Supervised and unsupervised setups were tested, validating the effectiveness and feasibility of the approach. The STM32 microcontroller is a common choice in the literature, and that's why ST Microelectronics has been personally involved in edge-AI research. El-Ouazzane (2024) have presented the novel architecture STM32N6 and the associated tool set (CubeAI). Such a solution provides similar AI performance to a quad-core processor with an AI accelerator, but at one-tenth the cost and one-twelfth the power consumption. Jouni et al. (2025a) has proposed a design framework capable of synthesizing a fully analog solution of a Multi-Layer Perceptron using TensorFlow tools and physics-informed models from post-layout transistor-level behavior. Such publications highlight that co-design is mandatory to address the trade-off between silicon area and energy efficiency for a specific NN architecture and training tools.

## 3 Spiking neural network

Neuromorphic circuits have gained significant attention in the literature as a potential bio-inspired solution for SNNs. In contrast to the MacCulloch and Pitts neuron model found in DNN implementations, SNNs seek biologically plausible and more complex mathematical models of neurons. Common choices are Hodgkin-Huxley (HH), Morris-Lecar (ML), Izhikevich, Resonate-and-Fire (R&F), Leaky Integrate-and-Fire (LIF), and Integrate-and-Fire (I&F), ordered from the most biologically inspired and complex to the simplest. In contrast with DNNs, SNN offers notable improvements in power efficiency and latency across various computational tasks. This is due to their event-driven and asynchronous nature, where processing elements communicate via spikes and consume energy only when active. Additionally, SNNs integrate memory and computation, thereby minimizing data transfer bottlenecks. Their spike-based (i.e., time- or rate-related) encoding can carry more information than traditional representations.

SpiNNaker (Furber et al., 2014) is one of the first projects to address the implementation of a large-scale SNN with 1,000 LIF neurons. Truenorth (Debole et al., 2019) takes brain-inspired processors to another level with 1 million LIF neurons and up to 256 million configurable synapses. Loihi 2 (Orchard et al., 2021) supports user-defined neuron models via programmable microcode, allowing for custom dynamics, including Izhikevich, R&F, or LIF. A single Loihi 2 chip supports up to 1 million neurons and roughly 120 million synapses, while multi-chip systems, such as Intel's Hala Point, support billions of neurons and synapses. However, digital neuromorphic circuits do not natively support biologically plausible models such as HH or ML. Another limitation is on power consumption, which makes them good competitors to cloud computing but not efficient enough for edge computing. Moradi et al. (2018) have proposed a hybrid analog/digital solution for I&F neuron models with asynchronous digital circuits, event-addressing traffic, and limited power consumption. Recently, Leone et al. (2025) has introduced a scalable edge-AI using a low-cost and low-power FPGA, also equipped with an RISC-V subsystem for flexible and reconfigurable SNN, theoretically supporting up to 65,000 neurons and 19 million synapses.

Analog neuromorphic circuits are usually spiking resonators, which are excited or inhibited by a control variable (i.e., current or voltage). Spiking rate and time (or phase) encoding are obtained from the physical phenomena of such a resonator. A decade of research was conducted before (Indiveri et al., 2011) consolidated the most common building blocks and techniques used to implement neuromorphic circuits. The Indiveri et al. (2011)'s experimental results from LIF and HH models have demonstrated the feasibility of ultra-low-power analog solutions, challenging digital ones in terms of higher energy efficiency. Sourikopoulos et al. (2017) have innovated on ML neuron design, highlighting the trade-off between speed and energy efficiency. The proposed ML (Sourikopoulos et al., 2017) and later LIF (Danneville et al., 2019) models have enabled a higher firing pattern operation, being one of the first publications on energy efficiency in the fJ/SOP range. Besrour et al. (2022) has designed a LIF neuron using conventional 28 nm technology, and the achieved performance evidences a promising solution for large-scale analog SNN. An edge solution using analog SNN is proposed by Jouni et al. (2023b), where an RF neuromorphic spiking sensor with an SNN of ML or LIF neurons is capable of recognizing the orientation of a transmitter.

Although SNNs offer a variety of learning algorithms, the efficient and well-established SGD learning algorithms from TensorFlow are not directly applicable. To overcome these limitations, Rioufol et al. (2023) has revisited the ML neuron from Sourikopoulos et al. (2017) in a novel way, able to deal with SNN limitations in deep learning through well-established algorithms. Such an ANN2SNN algorithm uses a non-spiking ANN, which is trained and then converted into an SNN. Wei et al. (2024) has developed the ANN2SNN conversion maps to train DNN activations into SNN firing rates or use backpropagation through time to directly optimize SNN temporal dynamics through surrogate gradients. Since there is no concept of time in ANNs, the ANN2SNN algorithm lacks temporal dependency, whereas both backpropagation through time (BPTT) and STDP algorithms are highly related to the timing of spike firing (Wei et al., 2024). The most widely used tool for SNN training is STDP due to its biological plausibility as a learning rule through the temporal correlation of events (Gautam and Kohno, 2021). STDP is a robust learning rule for SNNs, enabling on-chip unsupervised learning (Sun et al., 2022) and yielding excellent results in digital implementations. Nevertheless, Jouni et al. (2023a) has shown that noise significantly affects spike occurrence time in analog implementations due to transistor noise sources. Such work has suggested that the spiking rate could be a better metric in terms of noise immunity, while learning through spike timing may turn ω_*i, j*_ into a random variable, degrading SNN accuracy.

The lack of mainstream tools and the limitations in spike-timing representation led some authors to look for mathematical modeling solutions to represent ML and LIF behaviors as a non-linear *f*(·) comparable to RELU or sigmoid. Soupizet et al. (2023) have made an effort in considering physically informed *f*(·) in a synthesis framework based on TensorFlow, which revealed a mutually exclusive trade-off between deep learning and ultra-low power. Recently, Bossard et al. (2024) has demonstrated how the use of noise modeling in physically informed analog neuron models could improve SNN training and minimize the accuracy drop under noise. Moreover, Ferreira et al. (2025) have revealed how noise optimization of neuromorphic circuits could induce a stochastic resonance phenomenon, which may improve SNN performance under specific conditions. Khacef et al. (2023) have highlighted the trade-offs in neuron models, synaptic plasticity schemes, learning, and neural computation, providing a valuable survey on neuromorphic circuits and SNN training. Jouni et al. (2025b) have introduced an energy-efficient received-signal-strength SNN classifier for a 360° range and a 10° angular resolution relying on ML neurons and a customized training framework based on TensorFlow.

## 4 Discussion

**Figure 1:**
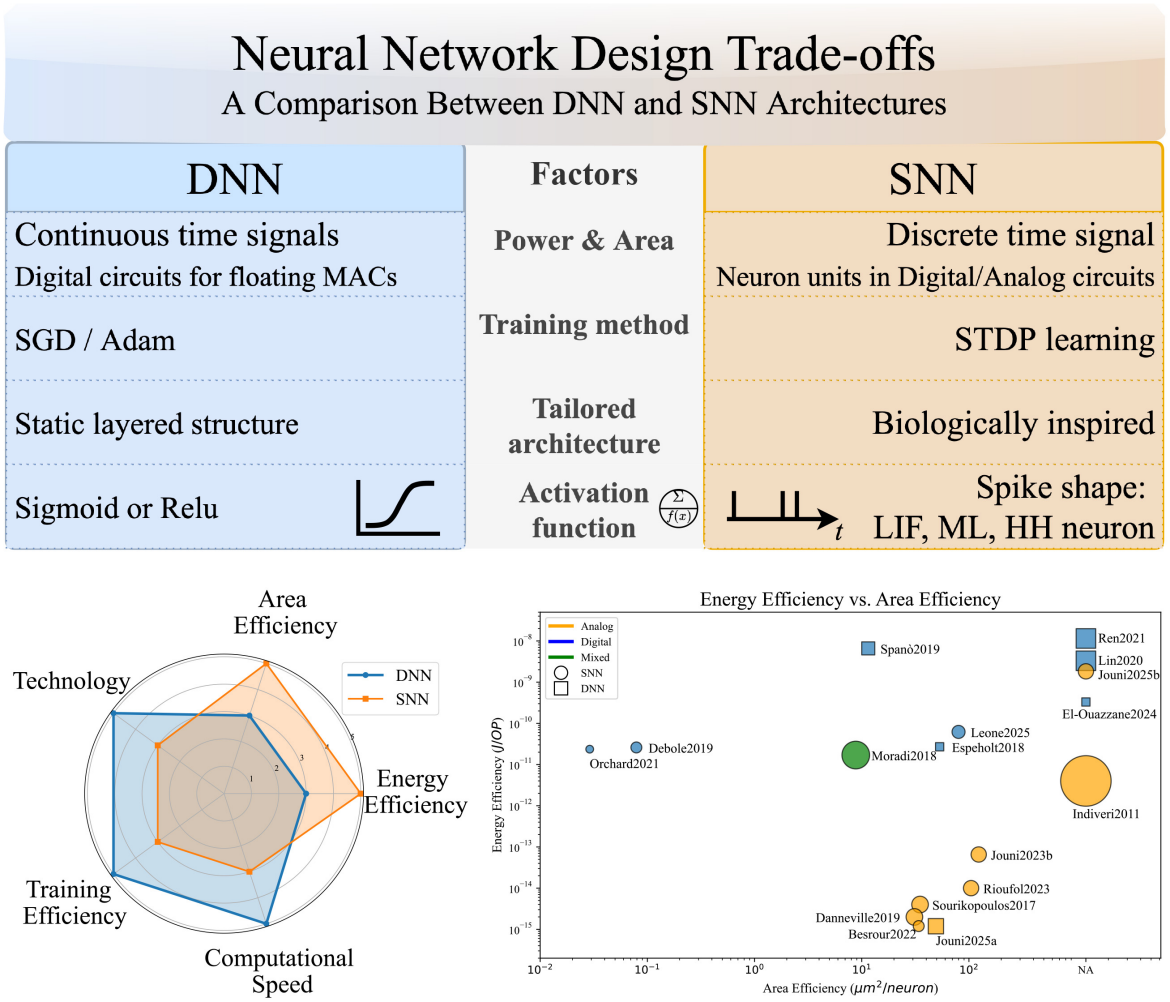
Neural network design trade-offs: a comparison between DNN and SNN architectures.

When implementing edge AI, designers often must choose between DNNs and SNNs. This decision involves balancing power consumption, hardware area, computational speed, and ease of training. Figure 1 illustrates the qualitative advantages and disadvantages of DNN and SNN implementations. DNNs are commonly deployed on general-purpose electronic circuits and process continuous-time signals, whereas SNNs require application-specific electronics, often called neuromorphic processing units (NPUs), to handle discrete-time signals. DNN operations depend on floating-point multiply-accumulate (MAC) units, which require large digital circuits (i.e., memory), whereas SNNs have neuron model units implemented in either digital or analog circuits. As a result, DNNs offer high-speed computation at the expense of significant power consumption and area requirements. Each SNN design is tailored to its spike model, limiting standardization and the availability of benchmarks.

DNNs benefit from well-established training algorithms, making them relatively easy to train. Ultimately, DNNs can autonomously learn hierarchical features from raw input data, eliminating the need for manual feature engineering. Nevertheless, training DNNs requires significant computational resources, including powerful GPUs and substantial memory. Their layered architectures rely on standard activations and can be limited by memory bandwidth. DNNs often require large, labeled datasets for effective training, which may not be available or adaptable for dynamic input streams. Moreover, DNNs exhibit robustness to noise and distortion in the input data, making them effective in real-world applications. In this context, the opaque nature of the decision-making process poses a challenge for creating reliable and explainable DNNs. DNNs can handle large-scale datasets and complex models, leveraging architectural versatility and scalability for improved accuracy. However, this is not without the risk of overfitting, considering the hyperparameters applied.

SNNs mimic the temporal dynamics and spike-based communication of biological neurons, enabling more realistic neural modeling. They are typically implemented on neuromorphic circuits, which have computation and memory on the same node (i.e., theoretically unlimited memory bandwidth). Analog and digital neuromorphic implementations face trade-offs between scalability, variability, and programmability. These neuromorphic circuits provide significant reductions in power and area compared to traditional DNNs. However, their computational speed is usually slower due to the asynchronous and event-based nature of spike communication. Effective learning algorithms for SNNs, like STDP, are less developed and more difficult to implement than SGD-based algorithms for DNNs. Compared to DNNs, SNNs currently lack standardized benchmarks and widespread practical applications. This encourages the development of tailored, physics-informed datasets for SNN evaluation. SNNs can model complex dynamics with low-power neuron models, especially suitable for sparse, event-driven, and spike-based applications, leading to potential energy savings and reduced redundant processing.

Figure 1 summarizes the major trade-offs discussed in the radar chart. See Figure 1 and the cited literature for detailed energy-area comparisons. Table 1 quantifies the figures of merit available in the literature. Energy efficiency is evaluated during NN inference, while power consumption is measured and normalized by the number of operating points (OP). Area efficiency is obtained from fabricated chip or layout estimations, normalized to the complexity of the NN model (i.e., number of neurons). One may observe that smaller technology nodes lead to better efficiency; this trend is consistent with Moore's Law.

**Table 1:** Literature comparison of neuromorphic circuits for edge-AI neural networks.

**References**	**Device architecture**	**Impl. model**	**Energy eff. (J/OP)**	**Area eff. (μm^2^/OP)**	**Tech**.
Indiveri et al. (2011) ^*^	Analog	HH	4 p	NA	0.6 μm
Sourikopoulos et al. (2017) ^*^	Analog	ML simpl.	4 f	35	65 nm
Moradi et al. (2018) ^*^	Mixed	I&F	17 p	8.8	0.18 μm
Espeholt et al. (2018) ^†^	Digital GPU	MAC	27 p	53.3	16 nm
Spanò et al. (2019) ^*^	Digital FPGA	MAC	6.6 n	11.5	40 nm
Danneville et al. (2019) ^*^	Analog	LIF	2 f	31	65 nm
Debole et al. (2019) ^*^	Digital NPU	LIF	26 p	0.079	28 nm
Lin et al. (2020) ^*^	Digital STM32F7	MAC	3.3 n	NA	90 nm
Ren et al. (2021) ^*^	Digital Arduino Nano 33BLE	MAC	11.5 n	NA	90 nm
Orchard et al. (2021) ^*^	Digital NPU NPU	Izhikevich, R& F or I&F	23.6 p	0.029	14 nm
Besrour et al. (2022) ^†^	Analog	LIF	1.2 f	34	28 nm
Jouni et al. (2023b) ^†^	Analog	ML, or LIF	65.5 f	123.4	55 nm
Rioufol et al. (2023) ^†^	Analog	ML bio	10 f	104.76	55 nm
El-Ouazzane (2024) ^†^	Digital STM32N6	MAC	330 p	NA	16 nm
Jouni et al. (2025a) ^†^	Analog	MPL	1.19 f	49.2	55 nm
Leone et al. (2025) ^*^	Digital FPGA	I&F	62 p	80	40 nm
Jouni et al. (2025b) ^†^	Analog	ML	1.83 n	NA	55 nm

Measurements ^*^; Simulations ^†^.

Analog architectures have a significant advantage in energy efficiency, consuming at least 1,000 times less power compared to digital ones in Sourikopoulos et al. (2017), Danneville et al. (2019), Besrour et al. (2022), Rioufol et al. (2023), and Jouni et al. (2025b). This advantage is due to weak inversion (sub-threshold) biasing, which is unavailable in digital solutions. Considering analog solutions, area efficiency may be ultimately limited at 30 μm^2^ by the relatively uniform capacitance density across integration technologies, like Sourikopoulos et al. (2017), Danneville et al. (2019), and Besrour et al. (2022). Digital architectures instead are not limited by the same factors and scale better in smaller nodes, like in Debole et al. (2019) and Orchard et al. (2021). DNNs developed in general-purpose electronics have lower device costs (development and production) than SNN competitors. Besides, edge-AI requirements are addressed by such hardware, like the Arduino Nano 33BLE from Ren et al. (2021) or the FPGA from Leone et al. (2025). Therefore, DNNs deliver superior computational performance and development convenience. They are limited by memory size and bandwidth while consuming more energy and silicon area.

SNNs provide a promising alternative for ultra-low-power and compact designs, although at the cost of slower operation and training complexity. The choice between these architectures presents a major trade-off in edge AI system design. In scenarios where fast response and robust training pipelines are essential, DNNs are often preferred. Conversely, for power-constrained or bio-inspired applications, SNNs offer compelling advantages. Computing and memory for SNNs are located within the same node, which requires a paradigm shift from von Neumann to neuromorphic computing. Indeed, SNNs will require problem-specific solutions. Frugal AI methods and physics-informed datasets are promising, since current general NN architectures lack SNNs.
